# Strontium Promotes Cementoblasts Differentiation through Inhibiting Sclerostin Expression *In Vitro*

**DOI:** 10.1155/2014/487535

**Published:** 2014-06-09

**Authors:** Xingfu Bao, Xianjun Liu, Yi Zhang, Yue Cui, Jindan Yao, Min Hu

**Affiliations:** ^1^Department of Orthodontics, School of Stomatology, Jilin University, 1500 Qinghua Road, Changchun, Jilin 130021, China; ^2^Institute of Virology and AIDS Research, First Hospital, Jilin University, Changchun 130021, China

## Abstract

Cementogenesis, performed by cementoblasts, is important for the repair of root resorption caused by orthodontic treatment. Based on recent studies, strontium has been applied for osteoporosis treatment due to its positive effect on osteoblasts. Although promising, the effect of strontium on cementoblasts is still unclear. So the aim of this research was to clarify and investigate the effect of strontium on cementogenesis via employing cementoblasts as model. A series of experiments including MTT, alkaline phosphatase activity, gene analysis, alizarin red staining, and western blot were carried out to evaluate the proliferation and differentiation of cementoblasts. In addition, expression of sclerostin was checked to analyze the possible mechanism. Our results show that strontium inhibits the proliferation of cementoblasts with a dose dependent manner; however, it can promote the differentiation of cementoblasts via downregulating sclerostin expression. Taking together, strontium may facilitate cementogenesis and benefit the treatment of root resorption at a low dose.

## 1. Introduction

Cementum is a type of special mineralized tissue lying on the surface of teeth roots and has an analogous chemical element constituent to bone [1, 2]. Due to its distinct position, cementum can connect with alveolar bone via fibers to maintain the position of teeth roots. Besides the function of roots anchoring, cementum is also believed to participate in the metabolic balance of dental tissues executed by cementoblasts [3–5]. This mentioned process is named as cementogenesis, which is often observed in periodontal tissue regeneration. One of such cases is the repair of root resorption origin from orthodontic treatment [6]. As a common adverse outcome in orthodontic treatment, root resorption usually occurred in more than 90% of orthodontic patients in clinic. Patients often endured root damage ranging from a slight loss of apical cementum to a total disappearance of root [7, 8]. When orthodontic force is unloaded, cementoblasts lying on the surface of roots start to repair the resorption lacuna. Although the etiology of root resorption is still unclear, cementum has been regarded as an essential part to prevent the resorption [9, 10]. What is more, cementogenesis has been well accepted as a target to alleviate root resorption currently. Thereby, various studies have been carried out to enhance the cementogenesis process [11–13].

As an effective antiosteoporosis drug, strontium ranelate can increase bone formation and inhibit bone resorption [14, 15]. Recent studies have demonstrated that strontium as the bioactive component of this drug could stimulate the proliferation and differentiation towards osteogenic direction in mesenchymal cells and osteoblasts [16, 17]. The mechanisms of strontium action are divided into calcium sensing receptor- (CaR-) dependent and CaR-independent signals. In the former one, strontium can act with CaR locating on the cell membrane and trigger different downstream pathways in osteoblast and osteoclast separately. Thus strontium can promote osteoblast replication, differentiation, and survival as well as reduce osteoclast differentiation, activity, and survival. In the latter one, some other signals such as Wnt signaling, OPG/RANKL signaling, and FGF/FGF receptor systems are involved [18]. Based on these dual activities on bone metabolism, strontium-contained drugs have been successfully applied in clinic to reduce the risk of fracture in osteoporosis patients and improve the bone healing [19, 20].

Growing bodies of evidences have indicated the characteristics similarity between osteoblasts and cementoblasts; however, some major differences are still existent between cementum and bone. For example, there is no typically vascular system and lamellar organization in cementum. Otherwise, biological regeneration is of absence for cementum [21]. These discrepancies indicated the potential differences in metabolism between cementoblasts and osteoblasts under similar periodontal environment. Thereby it is meaningful and interesting to clarify the response of cementoblasts towards the strontium, which will substantiate the feasibility of strontium for root resorption therapy. Herein, influence of strontium on the proliferation and differentiation of cementoblasts were checked in detail. At the same time, expression of sclerostin, an endogenous antagonist for Wnt signaling, was examined to investigate the possible mechanism involved.

## 2. Materials and Methods

### 2.1. Cell Culture

The immortalized murine cementoblasts cell line (OCCM-30) was a gift from professor Ding Bai (Sichuan University, Department of Orthodontics, Chengdu, China). In accordance with previous reports, OCCM-30 cells between 20 and 25 passages with fine capacity of differentiation were used in this research. The OCCM-30 cells were cultured in DMEM supplemented with 10% fetal bovine serum (FBS). The culture media contained 100 UI/mL penicillin and 100 UI/mL streptomycin. The cell lines were cultured in a humidified atmosphere containing 5% CO_2_ at 37°C.

### 2.2. Proliferation Analysis

Cells were seeded in 96-well plates at a density of 4 × l0^3^/well in *α*-MEM containing 1% FBS. After cell attachment, the media were changed to *α*-MEM with strontium of different concentrations. After 24 h incubation, 10 *μ*L MTT (5 g/L) was added to each well and cultured for another 4 h. Then supernatant was removed and 150 *μ*L DMSO was added. It was shaken for 15 min for the crystal dissolution. The absorbance at 490 nm was measured with a micro-ELISA reader (Synergy 2, BioTek, Winooski, VT, USA). The proliferation rate was calculated compared to the control group.

### 2.3. Staining of Cell

6 × l0^3^ cells per well were cultured overnight in 6-well plates and incubated together with strontium at 12.5 *μ*M for 24 h. Living cells were evaluated for morphology differences by employing calcein-AM staining. Generally, cells were washed twice with PBS (phosphate-buffered saline, pH 7.4) before calcein-AM (10 ng/mL) was added. After incubation for 20 min at 4°C in the dark, cells were observed using fluorescence microscopy (excitation (Ex), 365 nm and emission (Em), 480 nm).

### 2.4. Gene Analysis by Real-Time PCR

Cementoblasts cultured in 6-well plates were incubated with strontium at 12.5 *μ*M for 3 days. Total RNA was extracted using RNAiso Plus (Takara Co., Japan) according to the manufactures' instructions. Then RNA was analyzed for quality and quantity by measuring the A260/A280 ratio with ultraviolet spectrophotometry. 2 *μ*g of total RNA was used in following reverse transcription reaction by employing PrimeScript RT reagent kit (Takara Co., Japan). Then each sample was analyzed by quantitative real-time PCR (qPCR) (Stratagene MX3000P, Japan) in the SYBR Premix Ex TapII (Takara Co., Japan), setting the cycles as follows: 10 s/95°C PCR initial activation step, 40 cycles of denaturation for 20 s/95°C, and annealing step for 20 s/60°C. Formula 2^−(ΔΔCT)^ was used to determine the change in mRNA levels, where ΔCT is the value from the threshold cycle (CT) of the treated sample subtracted from the CT value of untreated or zero time-point control samples. Normalization to GAPDH mRNA was performed to decide the relative amount of mRNA in the sample. Primers used were listed in Table 1.

### 2.5. ALP Assay

Cells were seeded in 24-well plate at the density of 2 × 10^4^ cells/well. After cell attachment, the culture medium was changed to *α*-MEM, 10% FBS medium, and osteogenetic induction supplement containing 10 mmol/L disodium *β*-glycerophosphate and 0.15 mmol/L ascorbic acid. Strontium at 12.5 *μ*M was added to the culture medium of cells in treatment group. Then cells were incubated in the desired condition for 3 or 7 days followed by lysed using RIPA lysis buffer. Aliquots of supernatants were subjected to ALP activity and protein content measurement by an ALP activity kit and a microprotein assay kit (Jiancheng Biological Engineering Institute, Nanjing, China). ALP activities were normalized by total protein content.

### 2.6. Alizarin Red Staining

Cells were cultured in differentiation medium with or without 12.5 *μ*M strontium for 7 days. The formation of mineralized matrix nodules was determined by alizarin red staining. Briefly, the cells were fixed in 95% ethanol for 30 min at room temperature. After PBS washing, alizarin red (pH = 4.2) were added and incubated for 30 min at room temperature. Images of the staining results were recorded before quantitative analysis was performed. In detail, mineralization nodules were solved in 10% (w/v) cetylpyridinium chloride for 10 min at room temperature followed by measurement of OD at 570 nm. For mechanism analysis, recombinant human sclerostin (R&D, USA) at concentration of 30 ng/mL was added to the osteogenic medium containing strontium.

### 2.7. Western Blot

After incubation with or without strontium at 12.5 *μ*M for 7 days, 40 *μ*g of total protein was separated by SDS-PAGE, using a gradient gel ((10–12%), Bio-Rad Laboratories), transferred to nitrocellulose membrane, and analyzed by immunoblotting using the chemiluminescence (Santa Cruz, CA, USA). The primary antibodies used were sclerostin (Abcam, MA, USA, 1 : 500) or GAPDH (Santa Cruz, CA, USA, 1 : 1000), peroxidase-conjugated anti-mouse IgG (Santa Cruz, CA, USA, 1 : 1000). ImageJ software was applied to compare the intensity of protein bands to control through quantifying.

### 2.8. Immunofluorescence Microscopy

After incubation with or without strontium at 12.5 *μ*M, cells on coverslips were fixed with 10% formalin in phosphate-buffered saline (PBS), pH 7.3 for 10 min, and then permeabilized with 0.2% Triton X-100 in PBS for 5 min at room temperature. After blocking the cells with 1% skimmed milk for 1 h at room temperature, the cells were incubated with polyclonal mouse antisclerostin (Abcam, USA) antibodies at a 1 : 100 dilution overnight at 4°C. FITC-anti-mouse IgG (Santa Cruz, CA, USA) was used as a secondary antibody at a 1 : 200 dilution for 1 h at room temperature. Images were generated with a fluorescent microscope (BX 60, Olympus Co., Tokyo, Japan) operating a digital camera (DP 70, Olympus Co.).

### 2.9. Statistical Analysis

All experiments were performed thrice and the data were expressed as means ± SD. The difference between mean values was evaluated by using the ANOVA and considered to be statistically significant when *P* < 0.05.

## 3. Results 

### 3.1. Effect of Strontium Ions on the Proliferation of Cementoblasts

**Figure 1:**
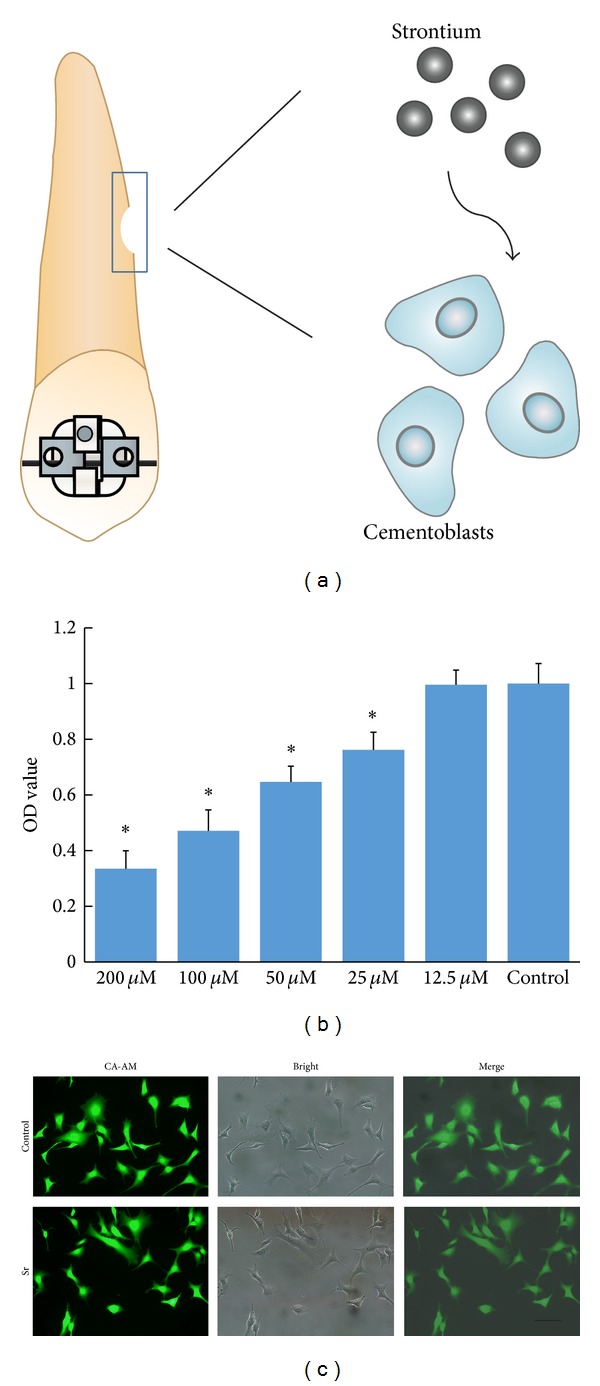
Schematic illustrating the modeling process of root resorption and the treatment of strontium (a). Cytotoxicity evaluated via MTT assays (b) and visible fluorescence microscopy images of OCCM-30 cells under different incubation conditions (c).

As shown in Figure 1(b), proliferation of cementoblasts is inhibited until the concentration of strontium ions decreased to 12.5 *μ*M.

### 3.2. Analysis for the Morphology of Cementoblasts

Cementoblasts exhibited the normal triangular form when incubated with 12.5 *μ*M of strontium ions. As shown in Figure 1(c), there is no obvious change between the control group and treatment group.

### 3.3. Gene Analysis

**Figure 2:**
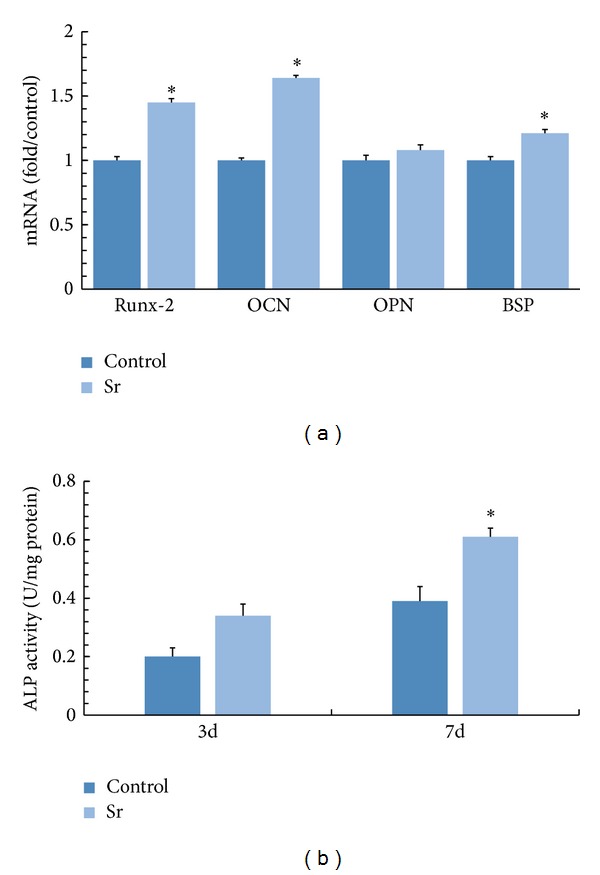
Expression of mineralization-related genes (a). ALP activity of cementoblasts after differentiation culture for 3 days and 7 days (b).

After cementoblasts were incubated with strontium ions for 3 days, gene expressions of Runx-2, OCN, and BSP were upregulated significantly (Figure 2(a)), but it is nearly the same for OPN expression between two groups.

### 3.4. Analysis for ALP Expression

After cementoblasts were cultured in the presence of strontium, ALP activity increased and became significant from day 3 to 7 after differentiation (Figure 2(b)).

### 3.5. Regulation of Sclerostin Expression by Strontium Ions

**Figure 3:**
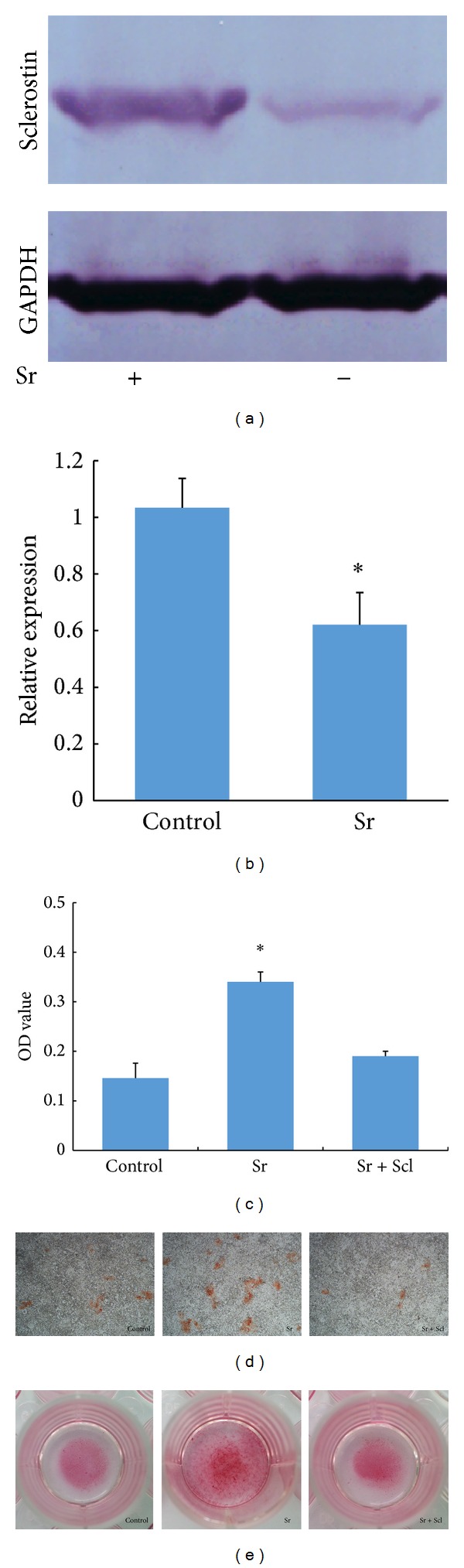
Expression of sclerostin after incubation with strontium ((a) and (b)). Alizarin red staining ((d) and (e)) and quantitative analysis (c) (Sr means strontium; Scl means recombinant human sclerostin).

**Figure 4:**
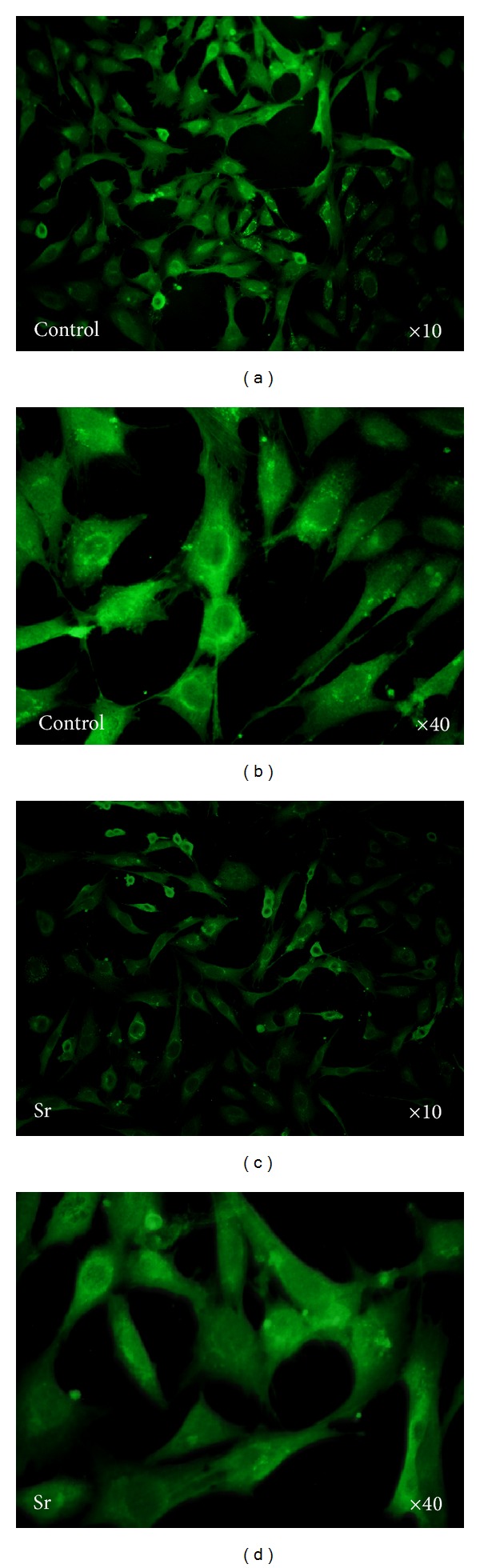
Immunofluorescence images of sclerostin in cementoblasts under different incubation conditions.

Result of western blot analysis showed that strontium ions inhibit the expression of sclerostin in cementoblasts (Figures 3(a) and 3(b)). The immunofluorescence staining images presented in Figure 4 also indicated the same trend.

### 3.6. Alizarin Red Staining

Addition of strontium ions to the medium promoted the mineralization of cementoblasts, which was partially attenuated by recombinant sclerostin (Figures 3(d) and 3(e)). As presented in Figure 3(c), the quantitative analysis of alizarin red staining revealed the same trend.

## 4. Discussions

As a common side effect of orthodontic treatment, root resorption often brings orthodontists into a dilemma in clinical practice. To extricate from the trouble, numerous methods have been tried to cure the resorption. Previous studies have shown that cellular cementum constitutes the main pattern of repaired roots, which is formed by cementoblasts originated from periodontal cell precursors [9]. Therefore it is important to clarify the regulatory mechanisms of cementogenesis induced by cementoblasts. In addition, agents used to benefit bone formation will also affect the metabolism of cementum due to the similarity between bone and cementum. Being a potential promoting agent to bone disease, little is known related to the function of strontium in cementum regeneration (Figure 1(a)). In current study, we proved the effect of strontium on cementoblasts proliferation and differentiation by inhibiting sclerostin expression.

The effect of strontium on the proliferation of cementoblasts was checked firstly. Cementoblasts were treated with diverse concentrations of strontium ranging from 12.5 *μ*M to 200 *μ*M for 24 hours. As shown in Figure 1(b), no significant change was observed at the concentration of 12.5 *μ*M. But when strontium concentration reached 200 *μ*M, only 35% of living cells were left compared to the control group, indicating the dose dependent inhibitory effect of strontium. This inhibition trend on proliferation is the same as previous studies performed on rat bone mesenchymal stem cells and osteoblasts [16, 22, 23]. But in some other reports, strontium is believed to promote cell proliferation at concentrations higher than ours [24], which may be due to the different cell type and methods used in these researches. Since strontium ranelate is oral administration and has influence on the whole body, it is necessary to pay attention to the potential toxicity. From the perspective of toxicity, dosage of strontium used for osteoporosis treatment should be limited. Alternatively, local administration of strontium can be considered for the dental related usage.

Based on the results of proliferation analysis, we selected the lowest 12.5 *μ*M concentration to perform other experiments. Living-cell staining by calcein-AM could provide more visible information. As illustrated in Figure 1(c), cementoblasts demonstrated triangular form with protuberances and there were no obvious differences between strontium treated group and control group. Now it is well accepted that normal proliferation and morphology are fundamental to cementogenesis, especially for cementum repair. Thereby, effect of strontium with suitable concentration on the proliferation of cementoblasts could provide new insight on strontium action.

Transcripts for Runx2, OCN, OPN, and BSP in the presence of strontium at concentration of 12.5 *μ*M were measured after 3 days incubation. As shown in Figure 2(a), the presence of strontium significantly altered the expression of Runx2, OCN, and BSP. All three gene expressions were upregulated against the control group. From the perspective of composition, cementum consists of 45%–50% hydroxyapatite and 50% collagen and noncollagenous matrix proteins [25]. Noncollagenous proteins existing in cementum include BSP, ALP, dentin matrix protein 1, osteopontin, and several growth factors. In common, Runx2 is regarded as a positive regulator of ALP, BSP, and OCN in the cementoblasts differentiation duration. OCN is a late marker for cementoblasts differentiation and participates in the mineral deposition. BSP, which is mainly lying on the root surface in cementogenesis, has been proved to trigger the mineralization and enhance the adhesion and differentiation of cementoblasts [26]. Therefore, gene analysis based on our study demonstrated that strontium might promote the differentiation of cementoblasts via upregulation of mineralization-related genes including Runx2, OCN, and BSP. As another important marker to describe the differentiation of cementoblasts, ALP expression was investigated after 3 days and 7 days of incubation. Figure 2(b) indicated that the ALP activity increased in the presence of strontium in a time-dependent manner. Alizarin red-S staining and OD value analysis were applied to evaluate the level of mineralization. As shown in Figures 3(c), 3(d), and 3(e), upon 7 days of incubation with strontium, red-stained mineralized nodules could be clearly observed from the control group and treated group. Highly in accordance with other differentiation markers, more red nodules could be seen in the presence of strontium.

To further clarify the possible mechanism involved in the positive effect on the differentiation of cementoblasts, we analyzed the expression of sclerostin after incubation of 7 days. As shown in Figures 3(a) and 3(b), sclerostin protein expression in cementoblasts occurred in the osteogenic differentiation culture medium. Compared with that of the control group, supplement of strontium into the differentiation medium could significantly reduce the expression of sclerostin by 30%. Otherwise, immunofluorescence analysis also indicated the above changes. In detail, stronger fluorescence could be observed in control group than in treated group (Figure 4). Encoded by SOST gene, sclerostin has been considered as an antagonist of Wnt signaling [27]. Because of the dual effect of sclerostin on bone turnover, the expression of sclerostin in dental tissue has attracted much more attention in recent years. For example, increasing the levels of sclerostin protein could be verified in the periodontal ligament cells after mineralization treatment [28]. In the present study, recombinant human sclerostin was further employed to investigate the speculation via the alizarin red staining. As described in Figures 3(c) and 3(d), enhanced mineralization induced by strontium could be shielded by the supplement of recombinant human sclerostin, indicating the inhibitory effect of sclerostin on the differentiation of cementoblasts [29].

## 5. Conclusions

In conclusion, we investigated the* in vitro* effect of strontium on the differentiation of cementoblasts via various experiments ranging from cell biology to molecular biology for the first time. Based on our results and other previous studies, one possible mechanism for above action was that strontium could inhibit the expression of sclerostin to promote the differentiation of cementoblasts. Thereby, strontium could be considered as a potential target to facilitate cementum repair in root resorption patients. However, due to the lower tolerance of cementoblasts towards strontium compared with osteoblasts and the complex environment* in vivo*, more studies were still highly needed before further application in clinic.

## Figures and Tables

**Table 1:** Primers used for quantitative real-time PCR.

Primers	Forward	Reverse
Runx-2	CTTCATTCGCCTCACAAAC	CTAGCAGTGACGGTCT
OCN	TGAACAGACTCCGGCG	GATACCGTAGATGCGTTTG
BSP	GAGACGGCGATAGTTCC	AGTGCCGCTAACTCAA
OPN	TTTACCAGCCTGCACCC	CTAGCAGTGACGGTCT
GAPDH	ACCACAGTCCATGCCATCAC	TCCACCACCCTGTTGCTGTA
